# Regulation of dermal fibroblasts by human neutrophil peptides

**DOI:** 10.1038/s41598-023-44889-8

**Published:** 2023-10-15

**Authors:** Nattarika Niwetbowornchai, Thanawat Chaisirirat, Sira Sriswasdi, Supichcha Saithong, Grace Filbertine, Helen L. Wright, Steven W. Edwards, Sita Virakul, Direkrit Chiewchengchol

**Affiliations:** 1 Center of Excellence in Translational Research in Inflammation and Immunology, Department of Microbiology, Faculty of Medicine, Chulalongkorn University, Bangkok, Thailand; 2 Center of Excellence in Immunology and Immune-Mediated Diseases, Department of Microbiology, Faculty of Medicine, Immunology Unit, Chulalongkorn University, Bangkok, 10330 Thailand; 3 Center of Excellence in Computational Molecular Biology, Chulalongkorn University, Bangkok, Thailand; 4 Research Affairs, Faculty of Medicine, Chulalongkorn University, Bangkok, Thailand; 5 Institute of Life Course and Medical Sciences, University of Liverpool, Liverpool, UK; 6 Institute of Infection, Veterinary and Ecological Sciences, University of Liverpool, Liverpool, UK; 7 Department of Microbiology, Faculty of Science, Chulalongkorn University, Bangkok, Thailand

**Keywords:** Computational biology and bioinformatics, Immunology, Molecular biology, Medical research, Molecular medicine

## Abstract

Human neutrophil peptides (HNPs) can induce cell proliferation and activation so their growth promoting activities may have potential clinical benefit. This study investigated the effects of HNPs on human dermal fibroblasts. Differential gene expression in HNP-treated cells and genes involved in regulating intracellular pathways were explored. Dermal fibroblasts were isolated from healthy neonatal foreskin and treated with HNPs in 2D and 3D cell culture systems. The expression of cell proliferation (*Ki-67*) gene and cell activation (*COL1A1*) gene plus their proteins was measured. Differential gene expression was determined using RNA-seq, and upregulated and downregulated genes were mapped onto intracellular pathways by KEGG analysis and Gene Ontology databases. HNPs significantly increased cell proliferation without cytotoxicity whilst HNP1 enhanced expression of *COL1A1* and type I collagen production in 2D cells and 3D spheroids. RNA-sequencing analysis showed gene clustering with clear separation between HNP1-treated and control groups. A heatmap of top 50 differentially expressed genes was consistent among HNP1-treated samples. Most upregulated genes were associated with cell proliferation and activation as mapped into intracellular pathways whilst most downregulated genes belonged to steroid/arachidonic acid metabolism and inflammatory signaling pathways. HNP1 increased cell proliferation and activation but reduced lipid metabolism and inflammation.

## Introduction

Human neutrophil peptides (HNPs) are members of the human α-defensin family and are classified as a group of antimicrobial peptides (AMPs). HNPs are produced by immune cells such as neutrophils and monocytes^1^. Different types of HNPs have been discovered and these peptides show a strong antimicrobial activity against various microorganisms^2,3^. However, HNPs play further roles in immune responses such as induction of cytokine production, pro- and anti-inflammation, and anti-tumor activity^4^.

Fibroblasts are the most abundant type of cells in dermis and provide structural integrity to the skin. The cells produce extracellular matrix particularly collagen which promotes tensile strength and elasticity^5^. For example, type I collagen produced by dermal fibroblasts is stacked, packed, and formed into collagen bundles that support the strength of the dermis^6–9^. Furthermore, dermal fibroblasts and inflammatory cells (e.g., neutrophils and macrophages) with their cytokines and AMPs are crucial for the first step of wound healing process^10^. Previous reports demonstrated that certain types of fibroblasts including dermal fibroblasts were activated after HNP stimulation^11,12^. However, studies of HNP-stimulated human dermal fibroblasts are very limited. Moreover, most studies demonstrated the action of HNPs in a conventional two-dimensional (2D) cell culture model which does not reproduce cell–cell interactions and tissue-specific architecture in the actual structure of human skin^13,14^.

Currently, three-dimensional (3D) cell culture systems have been developed and this model mimics skin structure and microenvironment as seen in vivo^15,16^*.* A cell cluster in a 3D model (e.g., spheroid) is generated by forming a complex of cell–cell adhesions (cell aggregation), and such models more realistic mimic in vivo conditions in terms of parameters such as gradient-dependent access of nutrients, gases and growth factors into the complex. Thus, spheroids in a 3D cell culture model reflects the microenvironment found in the actual cells and human tissues^14^.

In this study, the effects of HNPs on human dermal fibroblast proliferation and activation were investigated using 2D and 3D cell culture models as HNPs are released during wound healing process of the dermis^10^. We further identified changes of gene expression occurred in dermal fibroblasts after HNP stimulation by RNA sequencing and explored in depth the intracellular pathways in dermal fibroblasts using bioinformatic tools.

## Materials and methods

### Materials

The following reagents were used in this study: HNP1, HNP2 and HNP3 (Peptide Institute, Inc., Japan); TGF-β (BioLegend Inc., CA, USA); Dulbecco’s Modified Eagle’s Medium (Cytiva, Marlborough, MA, USA); Fetal Bovine Serum (Gibco, Grand Island, NY); ProLong™ Gold Antifade Mountant with DAPI (Invitrogen, CA, USA); LDH-Cytotoxicity Colorimetric Assay Kit II (BioVision Inc., CA, USA); RNeasy Mini Kit (QIAGEN Inc., Hilden, Germany); iScript Reverse Transcription Supermix, SsoAdvanced™ Universal Probes Supermix (Bio-Rad Inc., CA, USA); Pierce™ BCA Protein Assay Kit (Thermo Fisher Scientific Inc., NY, USA); 1X Protease/Phosphatase Inhibitor Cocktail, Rabbit anti-COL1A1 antibody, Mouse anti-Ki-67 antibody, Rabbit anti-β-actin antibody, Mouse anti-rabbit IgG antibody (HRP conjugate), Anti-rabbit IgG Alexa Fluor 555, Anti-mouse IgG Alexa Fluor 488 (Cell Signaling Technology Inc., MA, USA); Amersham ECL Western Blotting Detection Kit (GE Healthcare Life Sciences Inc., MA, USA); Alliance Q9 chemiluminescence imaging system (Uvitec Inc., UK); Tissue-Tek O.C.T.™ Compound (Sakura, Alphenaan den Rijn, Netherlands).

### Isolation and culture of dermal fibroblasts

Neonatal foreskin tissues were obtained by surgical circumcision of healthy male neonates at the Pediatric Surgery clinic, King Chulalongkorn Memorial Hospital with parental informed consent and assent forms. Ethical approval for this study was granted by the Institutional Review Board of the Faculty of Medicine, Chulalongkorn University (IRB 120/63). We confirm that all methods and experiments were performed in accordance with relevant guidelines and regulations. Dermal fibroblasts were isolated as described previously^17^ and cultured in medium containing DMEM supplemented with 10% FBS and gentamicin (1 mL/L). The cells were incubated in a 5% CO_2_ incubator at 37ºC, and the cells derived from the 2nd to 5th passage were used in experiments.

### Spheroid formation

Dermal fibroblasts (5 × 10^3^ and 1 × 10^4^ cells/well) in 100 µL of DMEM with 10% FBS were seeded into 96-well clear round bottom, ultra-low attachment plates. The medium was replaced with fresh medium every 3 days^18^. Spheroids were imaged at days 3, 5 and 7 and diameters were measured by ImageJ.

### Cell proliferation and cytotoxicity assays

Cell proliferation was analyzed by methylene blue staining. Dermal fibroblasts were seeded into a 96-well plate (3 × 10^3^ cells/well) with 1% FBS DMEM overnight. HNP1-3 (0.625–10 µM) were added into the wells, and the cells were incubated for 24 h. The supernatant was collected, and the cells were fixed with 20% (v/v) formaldehyde for 48 h and stained with methylene blue for 30 min. The cells were washed and eluted with 100 µL of ice-cold HCl (0.1 M) in absolute ethanol solution (1:1 ratio). The absorbance was measured at 650 nm using microplate reader. Cytotoxicity was analyzed using LDH-Cytotoxicity Colorimetric Assay Kit II. Collected supernatants (2.5 µL) were mixed with 25 µL of LDH reaction mix for 30 min. Stop solution (2.5 µL) was added and the absorbance was measured at 450 nm using microplate reader. Spheroids derived from dermal fibroblasts (5 × 10^3^ cells/well) were treated with HNP1-3 at 10 µM for 4 days. All experiments were performed in triplicates.

### RNA isolation and real-time PCR analysis

Dermal fibroblasts were seeded into a 6-well plate (2.5 × 10^5^ cells/well) in DMEM containing 1% FBS overnight. The cells were treated with HNPs (2.5, 5 and 10 µM) for 24 h. Total RNA was extracted and converted to cDNA with the following conditions: 25 °C for 5 min, 46 °C for 20 min and 95 °C for 1 min. *COL1A1* and *Ki-67* gene expressions were determined by real-time PCR. *ABL* gene expression was used as internal control. Primers and probes are listed in Supplementary Table S1 online. Real-time PCR was performed for 40 cycles with the following program: 95 °C for 2 min, 95 °C for 5 s and 60 °C for 30 s.

### Western blotting

Dermal fibroblasts were seeded into a 6-well plate (2.5 × 10^5^ cells/well) in 1% FBS in DMEM overnight. HNPs (2.5, 5 and 10 µM) were added into the wells and the cells were incubated for 48 h. Cells were lysed by 1X RIPA Lysis Buffer containing 1X Protease/Phosphatase Inhibitor Cocktail. Total protein concentration was measured by Pierce™ BCA Protein Assay Kit. Protein lysates (10 µg) were mixed with 2X SDS dye and heated at 100 °C for 5 min. Proteins were loaded in 7.5% SDS-PAGE and gel electrophoresis was performed at 100 V for 1.5 h. Proteins were transferred to PVDF membrane with electrophoresis at 15 V for 50 min. Blotting membranes were blocked with 1X PBS with 0.1% Tween-20 (PBST) containing 5% skimmed milk, followed by incubation with primary antibodies; COL1A1 (1:2000) and β-actin (1:4000), overnight at 4 °C. The membranes were washed with PBST, and mouse anti-rabbit IgG (HRP conjugate) secondary antibody (1:4000) was added. The membranes were incubated for 1 h with shaking before washing. The membranes were soaked in chemiluminescent substrate (Amersham ECL Western Blotting Detection Kit) and chemiluminescence signals were directly scanned with Alliance Q9 chemiluminescence imaging system. The band intensity was quantified by densitometry using ImageJ.

### Immunofluorescence staining

Dermal fibroblasts were seeded into a Lab-Tek II Chamber Slide System (1.5 × 10^4^ cells/well) in 1% FBS in DMEM overnight. HNPs (2.5, 5 and 10 µM) were added into the cells and incubated for 24 h. The cells were washed with PBS and fixed with 4% paraformaldehyde for 10 min. The cells were treated with 0.2% Triton- × 100 in PBS for 2 min and blocked with 1% BSA in PBS for 30 min. Primary antibody: Ki-67 (1:1000), diluted in 1% BSA in PBS was added and the cells were incubated at 4 °C overnight. After washing, secondary antibody: anti-mouse IgG Alexa Fluor 488 (1:2000), diluted in 1% BSA in PBS was added and the cells were incubated for 1 h. After washing, the sections were mounted and proteins were observed.

Spheroids derived from dermal fibroblasts (5 × 10^3^ cells/well) were treated with HNPs (10 µM) for 4 days. The spheroids were collected and covered with Tissue-Tek O.C.T.™ Compound. Frozen spheroids were cryosectioned into 8 µm thick layers onto glass slides. The sections were washed with PBS, fixed with 4% paraformaldehyde for 10 min and treated with 0.2% Triton- × 100 in PBS for 2 min. The sections were blocked with 5% BSA in PBS for 1 h and incubated with primary antibody: COL1A1 (1:400), diluted in 1% BSA in PBS at 4 °C overnight. After washing, the sections were incubated with secondary antibody: anti-rabbit IgG Alexa Fluor 555 (1:1000), diluted in 1% BSA in PBS for 1 h. After washing, the sections were mounted and proteins were observed.

### Statistical analysis

The statistical analyses were determined by paired t-test using GraphPad Prism 9.0.0 (GraphPad Software, Boston, MA, USA). A simple linear regression analyses was performed using STATA version 15.1 (StataCorp, College Station, TX USA). The regression coefficients, 95% confidence intervals (CI), and p-value were demonstrated. The results were expressed as the mean ± standard deviation (SD) and differences with a p-value < 0.05 were considered statistically significant.

### RNA-seq preprocessing and data analysis

Dermal fibroblasts were seeded into a 6-well plate (2.5 × 10^5^ cells/well) in DMEM containing 1% FBS overnight. The cells were treated with HNP1 (10 µM) for 24 h. Total RNA was extracted and the quality of extracted RNA (RNA Integrity Number ≥ 6.5) was evaluated using an Agilent 2100 Bioanalyzer. The RNA-seq experiment was conducted by Vishuo Biomedical, Thailand. Purified poly-A mRNA was fragmented, and pair-end RNA sequencing was performed on the Illumina HiSeq platform. The Gene Expression Omnibus (GEO) of raw reads in FASTQ files was GEO ID: GSE230670.

Quality of raw reads in FASTQ files was inspected with the FASTQC program (http://www.bioinformatics.babraham.ac.uk/projects/fastqc/). The Trim Galore program (http://www.bioinformatics.babraham.ac.uk/projects/trim_galore/) was used to cut adaptors and sequence reads with a Phred score lower than 30. To estimate abundance of transcript, cleaned raw reads were analyzed with Salmon v1.9.0^19^ by 2 steps; (1) indexing and (2) quantification. First, Salmon with default setting was used to build an index on human reference transcriptome (GRCh38) downloaded from Human Genome Resource at NCBI (downloaded; July 2022) (https://www.ncbi.nlm.nih.gov/projects/genome/guide/human/). Next, Salmon was used for quantification by mapping paired-end reads to the indexed reference sequence in mapping-based mode. Transcript abundances in estimated read counts were imported to R with tximeta v1.12.4^20^ and aggregated to gene-level expression with gene model annotation (GRCh38) for further analysis. Principal component analysis (PCA) was performed on the pre-processed gene expression data, which were first log-transformed and normalized with respect to library sizes by the rlog function in DESeq2^21^ package and standardized so that the expression level of each gene has a zero mean and a unit variance, to visualize the clustering structure of replicates. PCA plots were drawn in R using the ggplot2 package.

### Differential gene expression analysis

Differential gene expression was tested between HNP1 and control groups with DESeq2 v1.34.0^21^ package. Gene expression was normalized with the median of ratios method from DESeq2. Since samples were derived from different donors, statistical design for DESeq2 was accounted for donor factor when fitted generalize linear model to data. Multiple hypothesis testing correction was performed using Benjamini-Hochberg's procedure. Differentially expressed genes (DEGs) were defined as genes with false discovery rates (FDR) < 0.01. Boxplots were drawn in R using ggplot2.

### Functional enrichment analysis of gene set

Function of genes was analyzed with gene set enrichment analysis (GSEA) from WebGestalt (http://www.webgestalt.org/)^22^. The values of log fold changes were used to rank genes for the functional enrichment analysis using Gene Set Enrichment Analysis (GSEA) method. KEGG pathway and Gene Ontology databases (biological process, molecular function and cellular component) were used. Multiple hypothesis testing correction was performed using Benjamini-Hochberg’s procedure with the FDR cutoff of 0.05 for enriched functions.

## Results

### HNPs induced dermal fibroblast proliferation

**Figure 1:**
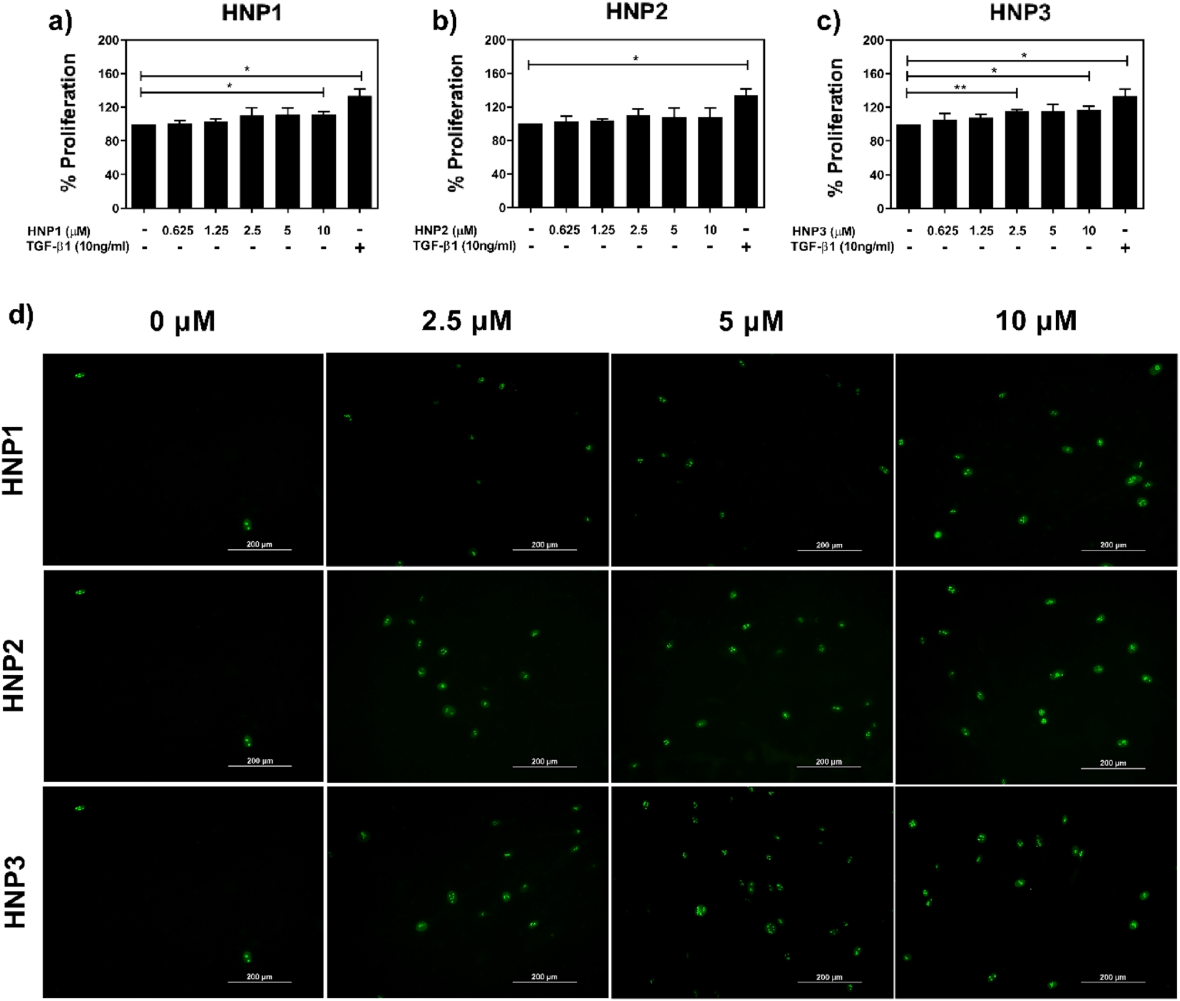
The effect of HNP1-3 on dermal fibroblasts. (**a**–**c**) The percentages of cell proliferation after dermal fibroblasts (n = 3) were treated with different concentrations (0.625–10 µM) of HNP1-3 for 24 h, measured by methylene blue staining (TGF-b1 as a positive control). (**d**) Representative immunofluorescence images of dermal fibroblasts stained with Ki-67 (green). Scale bars: 200 µm. (*P < 0.05 and **P < 0.01).

Cell proliferation and cytotoxicity were investigated after dermal fibroblasts were treated with different concentrations of HNP1-3. The results showed that HNP1 (10 μM), and HNP3 (2.5 and 10 μM) significantly increased cell proliferation (p < 0.05) (Fig. 1a–c). There is no detectable cytotoxicity at every concentration of HNP1-3 used (p > 0.05) (Supplementary Fig. S1a–c online). To determine the optimal concentration of HNPs on dermal fibroblast proliferation, the expression of *Ki-67* as a marker of dermal fibroblast proliferation was firstly investigated^23^. The results showed that both mRNA and protein expression levels of *Ki-67* were higher in cells treated with HNP1-3 (Fig. 1d) (Table 1). However, only HNP3 (5 and 10 μM) showed a statistically significant linear trend increase in *Ki-67* mRNA expression of dermal fibroblasts (p < 0.05).

**Table 1:** Summary of linear trend between HNP1-3 treatment to Ki67 protein and gene expression levels.

	Protein expression			Gene expression		
	Coefficient	p-value	95% CI	Coefficient	p-value	95% CI
HNP1_2.5uM	0.54	0.793	− 4.01, 5.08	1.57	0.153	− 0.73, 3.86
HNP1_5uM	0.90	0.660	− 3.65, 5.45	1.50	0.169	− 0.79, 3.80
HNP1_10uM	3.67	0.100	− 0.88, 8.22	1.40	0.197	− 0.90, 3.69
HNP2_2.5uM	3.80	0.373	− 5.50, 13.11	2.26	0.438	− 4.12, 8.65
HNP2_5uM	5.31	0.225	− 3.99, 14.61	3.75	0.213	− 2.64, 10.13
HNP2_10uM	4.36	0.311	− 4.94, 13.66	3.22	0.279	− 3.17, 9.60
HNP3_2.5uM	4.01	0.342	− 5.14, 13.15	1.12	0.062	− 0.07, 2.32
HNP3_5uM	5.97	0.171	− 3.18, 15.11	2.33	0.002*	1.14, 3.52
HNP3_10uM	4.26	0.314	− 4.89, 13.40	2.49	0.001*	1.13, 3.68

*P < 0.05

### HNPs activated production of type I collagen in dermal fibroblasts

**Figure 2:**
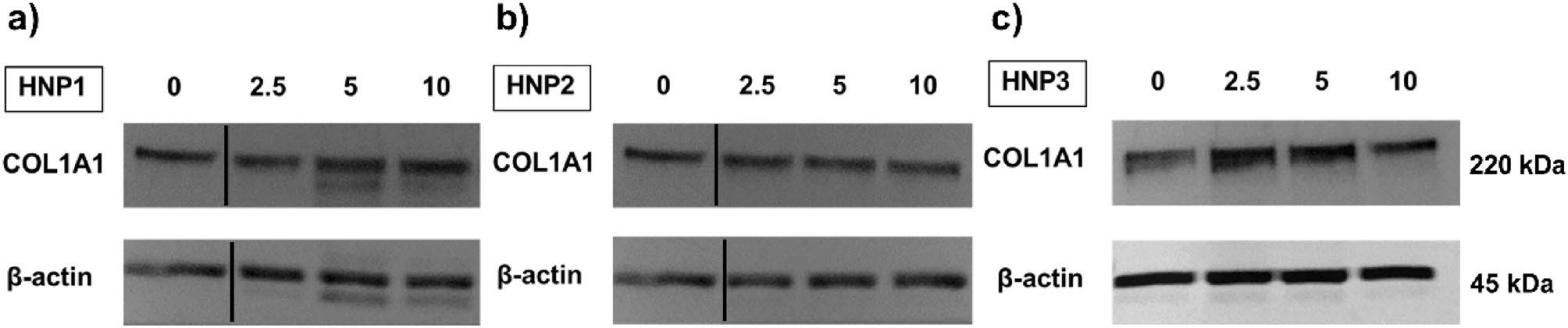
The effect of HNP1-3 on dermal fibroblast activation. (**a**–**c**) The protein expression of collagen type I and representative images of band intensity after dermal fibroblasts (n = 3) were treated with HNP1-3 at the concentrations of 2.5, 5 and 10 µM for 48 h (Cropped blots are displayed with dividing lines. The original blots are presented in Supplementary Fig. 5).

Next, we investigated the optimal concentration of HNPs on dermal fibroblast activation. Type I collagen is the most abundant type of collagen in dermis which is expressed by activated dermal fibroblasts^24^. The results showed that HNP1-3 increased expression of *COL1A1* gene and only HNP1 (5 and 10 μM) significantly enhanced *COL1A1* gene expression (p < 0.05) (Fig. 2a–c) (Table 2). Moreover, protein expression of type I collagen was increased in cells treated with HNP1 (5 and 10 μM). HNP3 (10 μM) significantly increased *COL1A1* gene expression (p < 0.05).

**Table 2:** Summary of linear trend between HNP1-3 treatment to type I collagen protein and gene expression levels.

	Protein expression			Gene expression		
	Coefficient	p-value	95% CI	Coefficient	p-value	95% CI
HNP1_2.5uM	0.21	0.111	− 0.59, 0.47	0.22	0.268	− 0.21, 0.64
HNP1_5uM	0.36	0.013*	0.10, 0.63	0.43	0.049*	0.01, 0.85
HNP1_10uM	0.37	0.013*	0.10, 0.63	0.66	0.007*	0.23, 1.08
HNP2_2.5uM	0.42	0.172	− 0.23, 1.07	0.25	0.437	− 0.46, 0.96
HNP2_5uM	0.26	0.389	− 0.39, 0.90	0.63	0.074	− 0.08, 1.35
HNP2_10uM	0.46	0.139	− 0.19, 1.11	0.56	0.107	− 0.15, 1.27
HNP3_2.5uM	0.17	0.300	− 0.18, 0.53	0.65	0.115	− 0.20, 1.51
HNP3_5uM	0.14	0.393	− 0.22, 0.49	0.56	0.169	− 0.29, 1.41
HNP3_10uM	-0.05	0.748	− 0.41, 0.30	0.90	0.042*	0.04, 1.75

*P < 0.05

**Figure 3:**
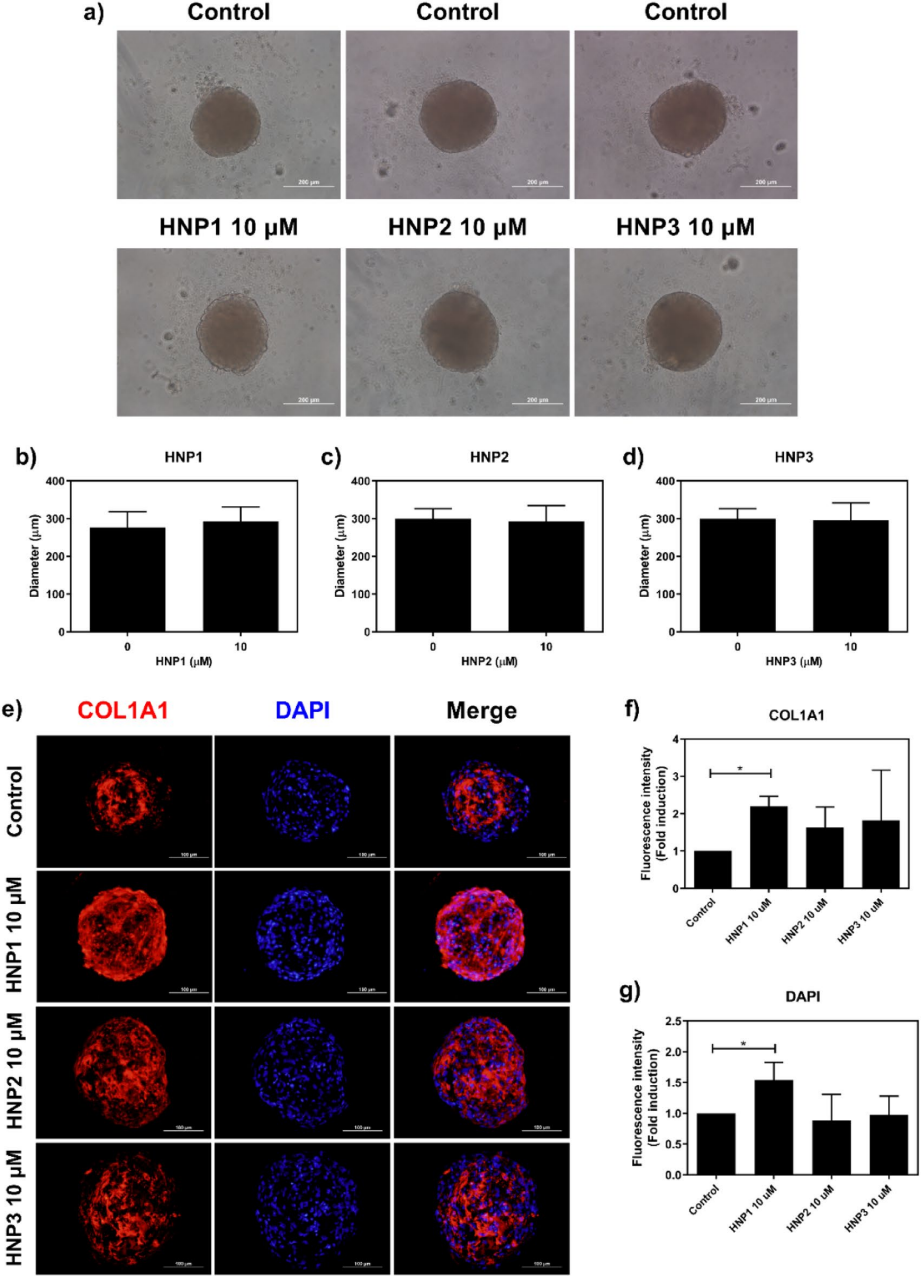
The effect of HNP1-3 on spheroids of dermal fibroblasts. (**a**) Representative images of spheroids treated with HNP1-3 (10 µM) under light microscope (n = 3) and (**b**–**d**) diameters. Scale bars: 200 µm. (**e**) Representative immunofluorescence images of spheroids stained with COL1A1 (red) and DAPI (blue). Scale bars: 100 µm. Mean fluorescence intensity of (**f**) COL1A1 and (**g**) DAPI detected after spheroids were treated with HNP1-3 (10 µM). (*P < 0.05).

We further investigated the expression of type I collagen in Spheroid 3D cell culture system. Spheroid formation of dermal fibroblasts was successfully generated with approximate diameters of 300–400 µm (Supplementary Fig. S2a,b online), and HNPs did not interfere the spheroid formation in terms of shape and diameter (Fig. 3a–d). The results showed that all HNP1-3 (10 μM) increased expression of type I collagen (Fig. 3e), particularly HNP1 that showed the strongest expression of type I collagen and DAPI (Fig. 3f,g). This observation was confirmed by a significant increase of type I collagen and DAPI in spheroids treated with HNP1 (p < 0.05).

### RNA-Seq and differential gene expression analyses

**Figure 4:**
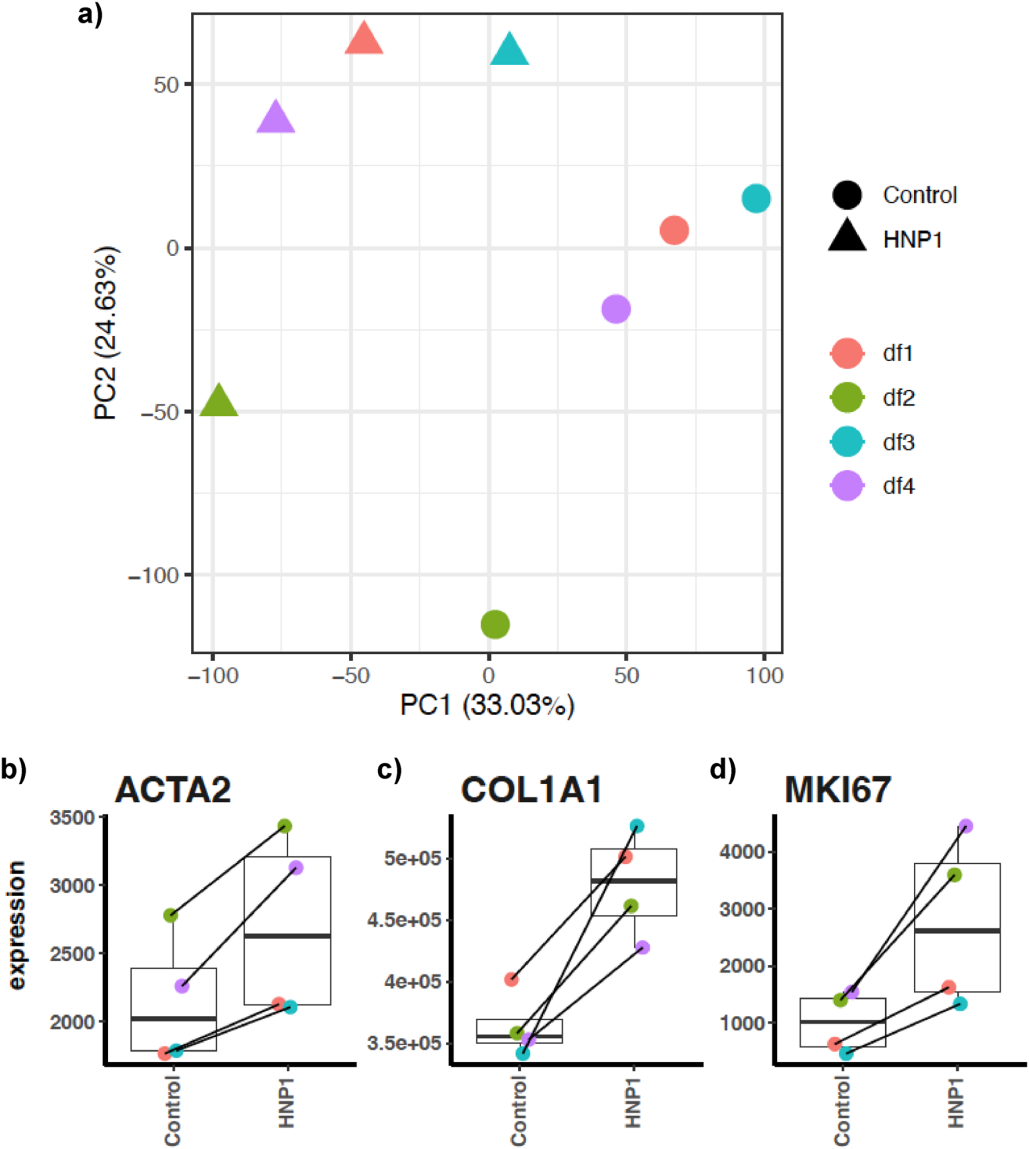
(**a**) Principal component analysis (PCA) of standardized gene expression data. The first principal component (PC1) separates HNP1-treated and control groups. The second principal component (PC2) separates other samples from df2. (**b-d**) Scatter plots comparing normalized read counts between HNP1-treated and control groups for *ACTA2*, *COL1A1* and *MKI67*.

As HNP1 demonstrated significant increases in both cell proliferation and activation compared to other HNPs, we therefore investigated differential gene expression and intracellular pathways after dermal fibroblasts were treated with 10 μM of HNP1 using RNA-sequencing. On average, 16–32 million reads were acquired and more than 92% could be mapped to reference transcripts (Supplementary Table S2 online). Principal component analysis (PCA) of standardized read counts showed clustering of biological replicates with consistent separation between HNP1-treated and control groups along the direction of the first principal component (Fig. 4a). The second principal component separates one donor (df2) from the others. Next, we focused upon the expression levels of genes involved in cell proliferation and activation. The results showed that *COL1A1* expression was upregulated by 1.22–1.55 fold whilst *MKI67* (Ki-67) expression was upregulated by 2.58–2.90 fold and *ACTA2* (α-SMA) expression was upregulated by 1.18–1.39 fold (Fig. 4b–d).

Differentially expressed genes (DEGs) between HNP1-treated and control groups were identified using DESeq2 with an FDR cutoff < 0.01. In total, there were 3,734 DEGs with 1,877 upregulated genes (51%) and 1,857 downregulated genes (49%). Heatmap of the top 50 DEGs with the highest absolute log2 fold changes (HNP1-treated/control) was shown in (Supplementary Fig. S3 online).

### Functional enrichment analysis

**Figure 5:**
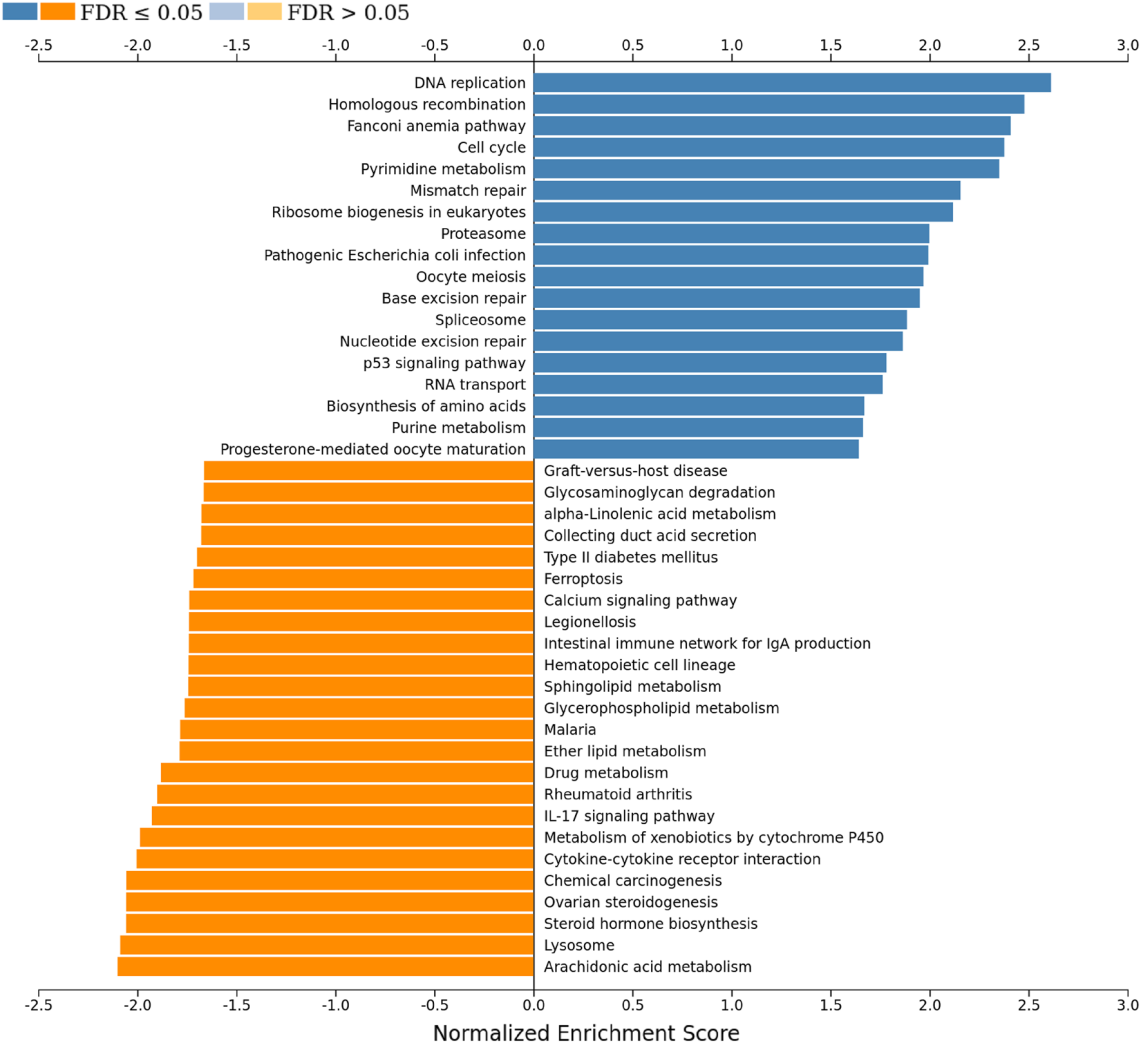
Enriched KEGG functional terms among DEGs. Positive enrichment corresponds to upregulation of gene expression levels in the HNP1-treated condition. Negative enrichment corresponds to downregulation. Image was generated from WebGestalt with all enriched terms with FDR < 0.05.

**Figure 6:**
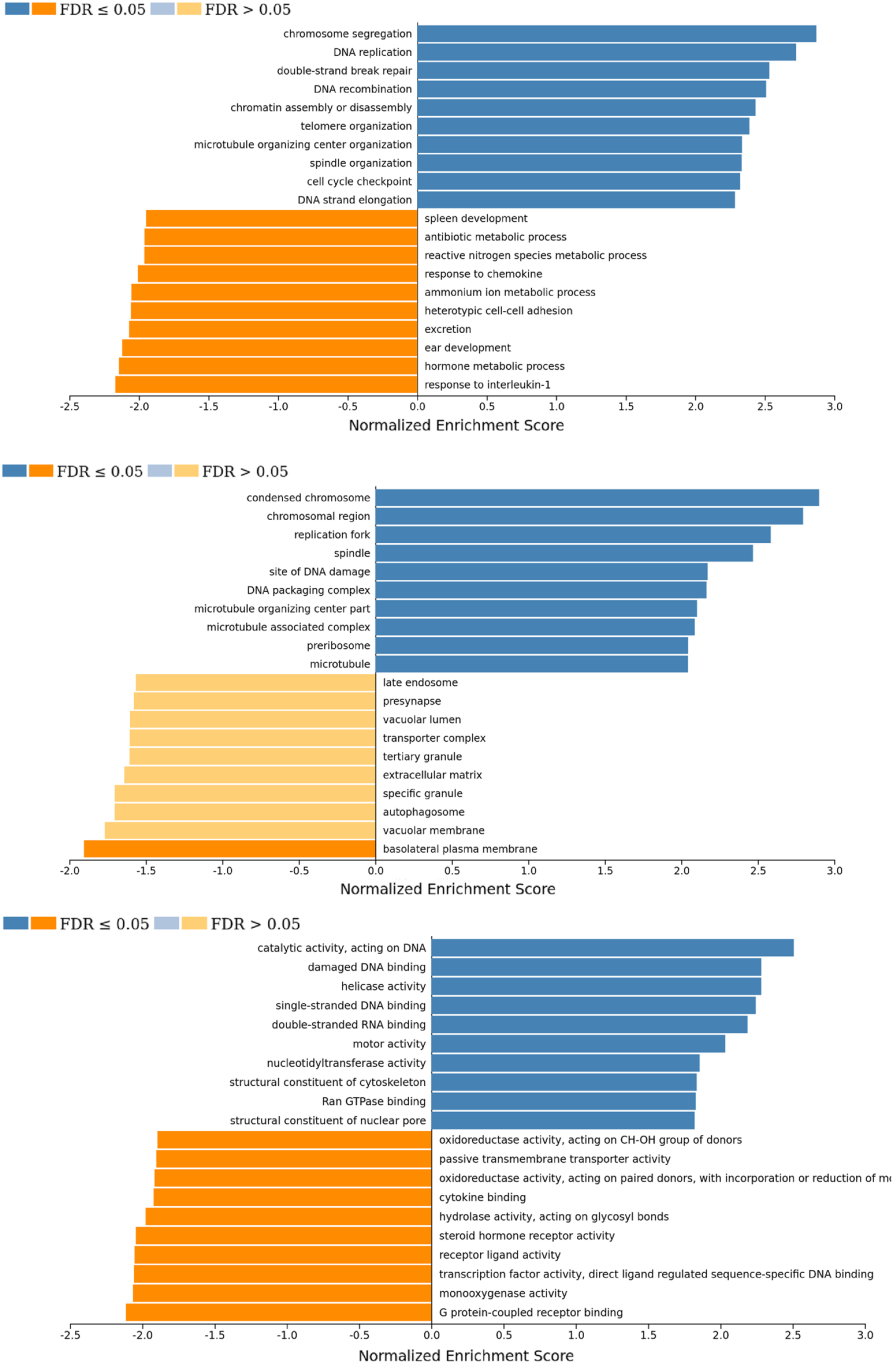
Bar plot of normalized gene set enrichment score from differentially expressed genes (DEGs) analyzed with gene set enrichment analysis (GSEA) in Gene Ontology database; (**a**) biological process (**b**) cellular component and (**c**) molecular function categories.

We determined intracellular pathway activation in HNP1-treated dermal fibroblasts based on changes in gene expressions. Gene set enrichment analysis (GSEA) was performed by ranking genes with log2 fold changes. The result showed that 18 upregulated pathways and 24 downregulated pathways were identified as enriched categories (Fig. 5). Gene Ontology databases showed upregulation of DNA replication, DNA damage repair, and cell cycle in HNP1-treated groups (Fig. 6). The expression profiles of genes in these pathways were consistently upregulated or downregulated in all samples (Supplementary Fig. S4 online).

## Discussion

Dermal fibroblasts provide strength and elasticity of human skin^25^. After skin injury, dermal fibroblasts proliferate and produce extracellular matrix (e.g. type I collagen) to regenerate the skin structure and initiate the process of wound healing. Several factors are involved in the wound healing process such as epithelial cells, cytokines including antimicrobial peptides^4,26^. Recent studies have demonstrated that antimicrobial peptides not only eliminate microorganisms but also stimulate different cell types^4^.

In this study, we determined the effects of HNP1-3 on the proliferation and activation of human dermal fibroblasts. First, our study demonstrated that HNP1 significantly increased cell proliferation (Fig. 1a), which was consistent with previous studies demonstrating a proliferative effect of HNP1 on mouse fibroblasts, as well as human conjunctival and lung fibroblasts^11,27–29^. However, the effect of HNP2 on cell proliferation was not observed in our study (Fig. 1b), probably because of cell-type specificity, because a previous study reported that lung fibroblast proliferation was significantly increased after HNP2 treatment^29^. We found that HNP3 significantly increased cell proliferation (Fig. 1c) which has never been reported. Moreover, a cytotoxic effect of HNP1-3 (up to 10 μM) on dermal fibroblasts was not observed in our study (Supplementary Fig. S1a–c online).

Next, we investigated expression of key genes in dermal fibroblasts, firstly *Ki-67*, which is involved in dermal fibroblast proliferation. The results demonstrated that all HNPs increased levels of *Ki-67* mRNA expression although the effects of HNP1-and HNP2-treated cells did not reach statistical significance, probably due to biological variations in our primary tissue samples. The increased expression of *Ki-67* was supported by the enhanced protein expression of Ki-67 although again increases did not reach statistical significance (Fig. 1d) (Table 1).

Dermal fibroblasts become activated by different factors, such as fibroblast growth factors and cytokines (e.g. TGF-β), and produce extracellular matrix especially type I collagen to restore tissue integrity during the wound healing process^30,31^. We investigated the expression of both *COL1A1* gene and type I collagen protein after dermal fibroblasts were activated with HNPs. The result showed that HNP1 (5 and 10 μM) and HNP3 (10 μM) significantly increased the expression of *COL1A1* gene which was consistent with previous studies^12,28,29^. However, HNP1 (5 and 10 μM) showed significantly increases in type I collagen protein expression (Fig. 2a–c) (Table 2). These findings were consistent with previous studies demonstrating that HNP1 increased collagen synthesis in activated dermal fibroblasts, conjunctival fibroblasts and lung fibroblasts^11,12,28,29^. Notably, the concentrations used in our study were different from the previous studies suggesting that microenvironment which may contain varying concentrations of HNPs can specifically affect cells and tissues in different organs.

3D-cell culture systems (e.g. spheroids) have been developed in order to imitate more closely the true microenvironment of human fibroblasts^15,16^. Spheroid formation is reproducible and commonly used in many studies especially for investigations on pharmacological effects^32,33^. However, spheroid formation may decrease cell viability because of limitations in nutrient sources and oxygen supply (normoxia at the periphery and hypoxia at the center of spheroid), particularly in spheroids with larger diameter (> 500 µm)^34–36^. In this study, spheroids from dermal fibroblasts were generated with approximate diameters of 300–400 µm (Supplementary Fig. S2 online). We investigated the effects of HNP1-3 on the spheroids and found that all HNPs did not impede spheroid formation nor alter spheroid diameter (Fig. 3a–d). On the contrary, HNPs increased cell proliferation (DAPI staining) and type I collagen (COL1A1) in these spheroids (Fig. 3e–g), particularly HNP1 which demonstrated the highest increases in DAPI staining and type I collagen expression. These findings indicate that HNP1 is potentially a good candidate for treatment of dermal fibroblast proliferation and activation.

To gain further insights into the effect of HNP1, we investigated the expression of genes involved in intracellular pathways in HNP1-treated cells using RNA-sequencing. The DEGs and enriched predicted biological functions showed a clear separation between HNP1-treated cells from control group (Fig. 4a). However, there was variation in gene expression profiles (donor ID: df2), probably because of differences in the underlying biological status. Next, we determined the expression levels of common genes involved in cell proliferation and activation of dermal fibroblasts; *COL1A1*, *MKI67* (Ki-67) and *ACTA2* (α-SMA; fibroblast activation and contractility)^31,37,38^, and found increased expression levels of these genes in all samples (Fig. 4b–d). Although these genes were not in the top upregulated genes and the overall gene expression profiles of samples from df2 differed from the others, the heatmap of top DEGs (Supplementary Fig. S3 online) including the expression patterns of *COL1A1*, *MKI67* and *ACTA2* were consistent among all samples. This observation supports the experimental evidence that HNP1 induces cell proliferation and activation of dermal fibroblasts. Moreover, HNP1 induced upregulation of genes associated with cell proliferation and activation as the majority of these upregulated genes were mapped into intracellular pathways of a) DNA replication (e.g. pyrimidine metabolism, ribosome biogenesis, biosynthesis of amino acids), b) DNA damage repair (e.g. mismatch repair, base excision repair, nucleotide excision repair), and c) cell cycle (Figs. 5, 6 and Supplementary Fig. S4 online). In contrast, most individual downregulated genes belong to various pathways such as lysosome, steroid hormone biosynthesis, arachidonic acid metabolism and IL-17 (Fig. 5 and Supplementary Table S3 online). This evidence suggests that HNP1 decreases lipid (steroid and arachidonic acid) metabolism and inflammation^39,40^ but drive dermal fibroblasts towards cell proliferation and activation including collagen production. However, other individual upregulated and downregulated genes belong to various pathways with no clear correlation between these events that may explain functional changes. In addition, the receptor for HNP1 binding to dermal fibroblasts is unknown and therefore identification of these mechanisms will further delineate the signaling pathways responsible for its effects on proliferation and activation.

In conclusion, this study demonstrated that HNP1-3 induced cell proliferation and activation of human dermal fibroblasts without cytotoxicity but only HNP1 increased both cell proliferation and type I collagen production at higher levels than other HNPs. One limitation of our study is the small sample size and biological variations among samples. Despite this, the effects of HNP1 were confirmed using RNA-sequencing analysis and demonstrated upregulated genes involved in cell proliferation and type I collegen expression. Furthermore, the most upregulated genes were mapped into intracellular pathways such as DNA replication, DNA damage repair and cell cycle. We propose that HNP1 could potentially be used as an adjunctive treatment for wound healing and other dermatological conditions such as skin aging that needs dermal fibroblast activation and type I collagen synthesis.

### Supplementary Information

Supplementary Information.

## Data Availability

The authors confirm that the data supporting the findings of this study are available within the article [and/or] its supplementary materials. The datasets generated and/or analysed during the current study are available in the Gene Expression Omnibus under accession number GSE230670.
