# Supplementary material for: Regulation of dermal fibroblasts by human neutrophil peptides
Source: Sci Rep. 2023 Oct 15;13:17499. doi: 10.1038/s41598-023-44889-8 (PMC10577140; doi:10.1038/s41598-023-44889-8)
Supplement: Supplementary file 1 — Supplementary Information. [file 41598_2023_44889_MOESM1_ESM.docx]

**
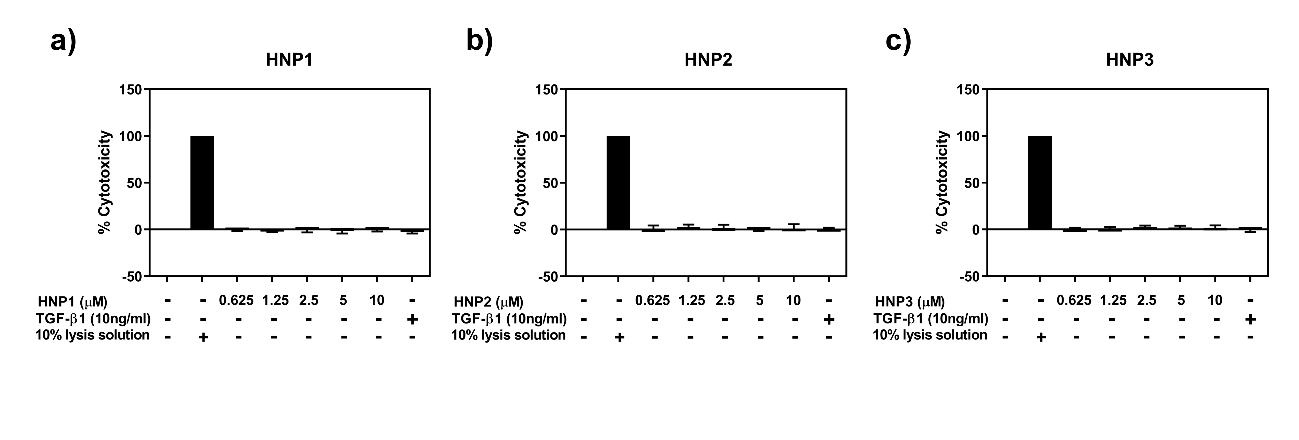
**

**Supplementary Figure 1**. The effect of HNP1-3 on dermal fibroblasts. **(a-c)** The percentages of cytotoxicity after dermal fibroblasts (n=3) were treated with different concentrations (0.625-10 µM) of HNP1-3 for 24 h, measured by LDH-Cytotoxicity Assay (10% lysis solution as a positive control).


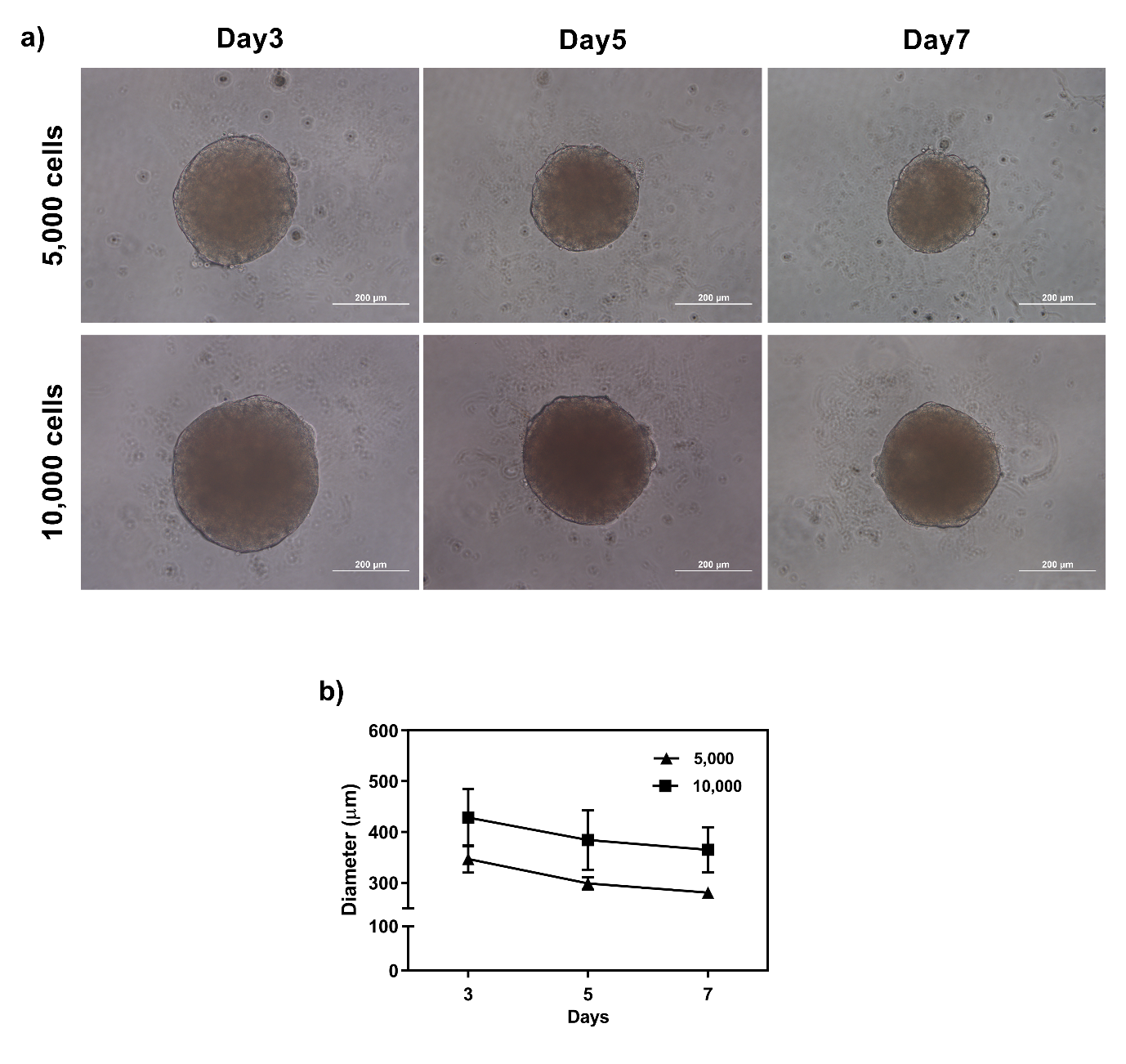


**Supplementary Figure 2.** Spheroid formation of dermal fibroblasts. **(a)** The spheroids were initially observed under light microscopy at day 3, 5 and 7. Representative images of spheroids from two concentrations of dermal fibroblasts (5,000 and 10,000 cells/well). Scale bars: 200 µm. **(b)** The diameters of spheroids from dermal fibroblasts measured by ImageJ. Data represented as mean ± SD (n=3)


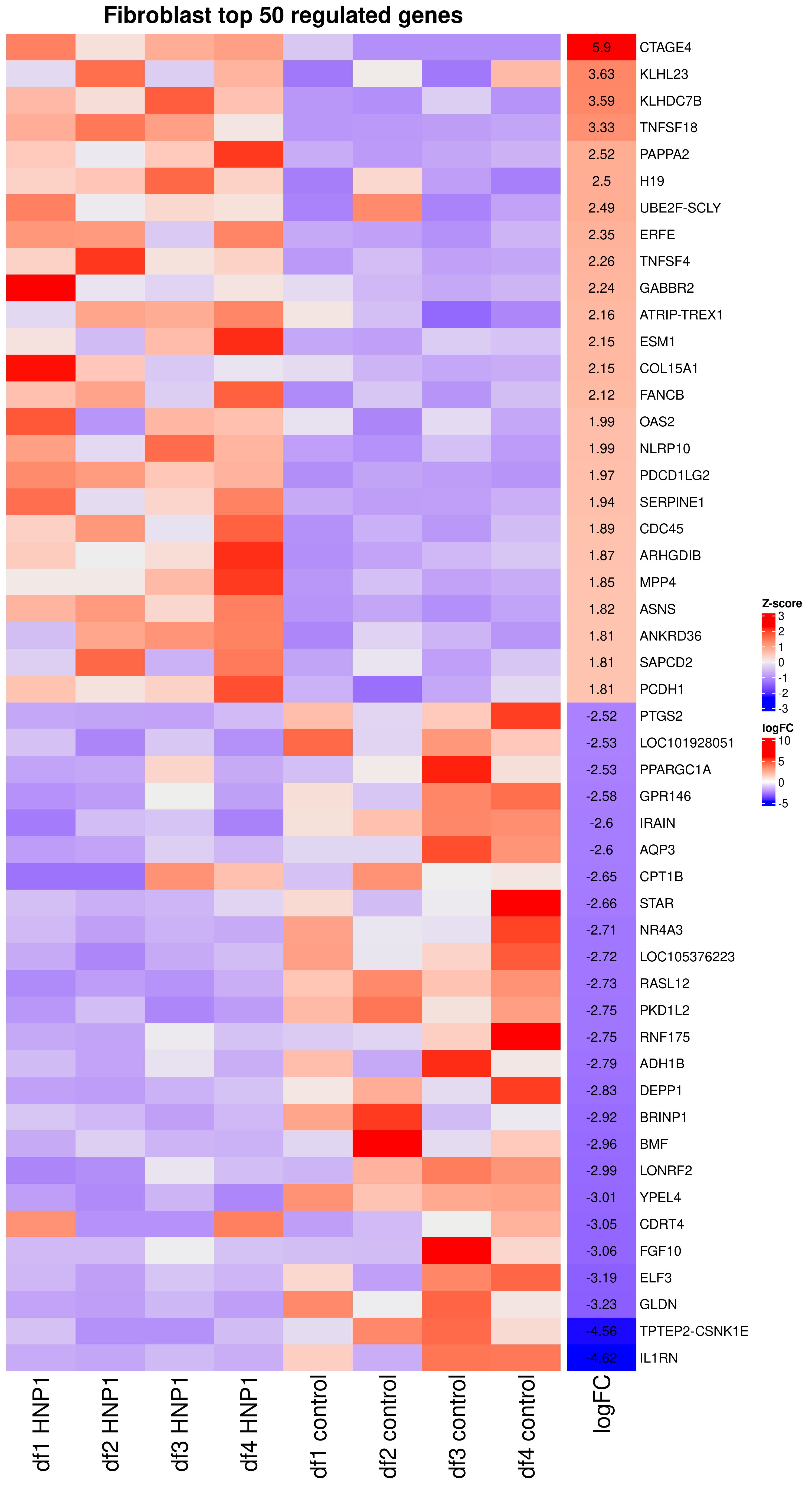


**Supplementary Figure 3**. Heatmap of gene expression levels for the top 50 DEGs. Genes were ranked using the average values of log fold changes across samples (25 genes for each upregulated and downregulated genes). Heat map image was generated using ComplexHeatmap package version 2.16.0 (<https://bioconductor.org/packages/release/bioc/html/ComplexHeatmap.html>) in R version 4.3.1


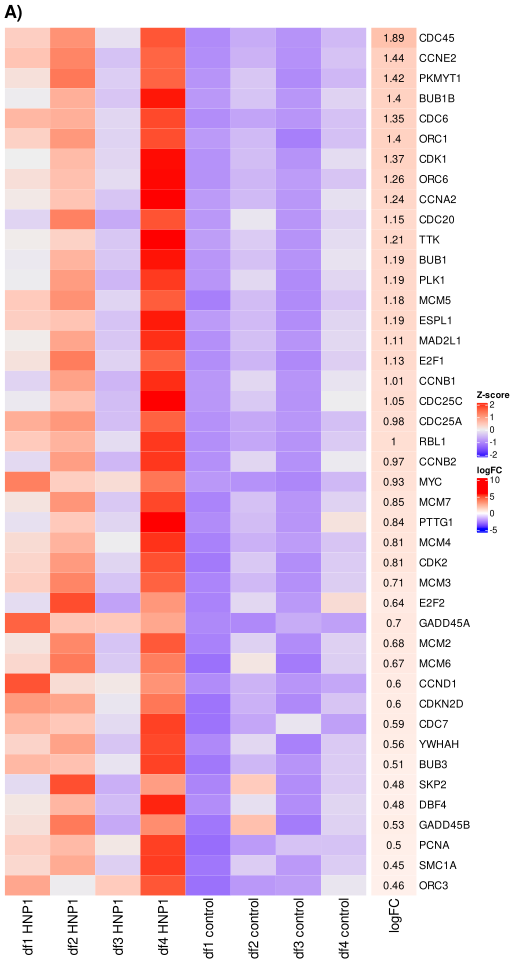

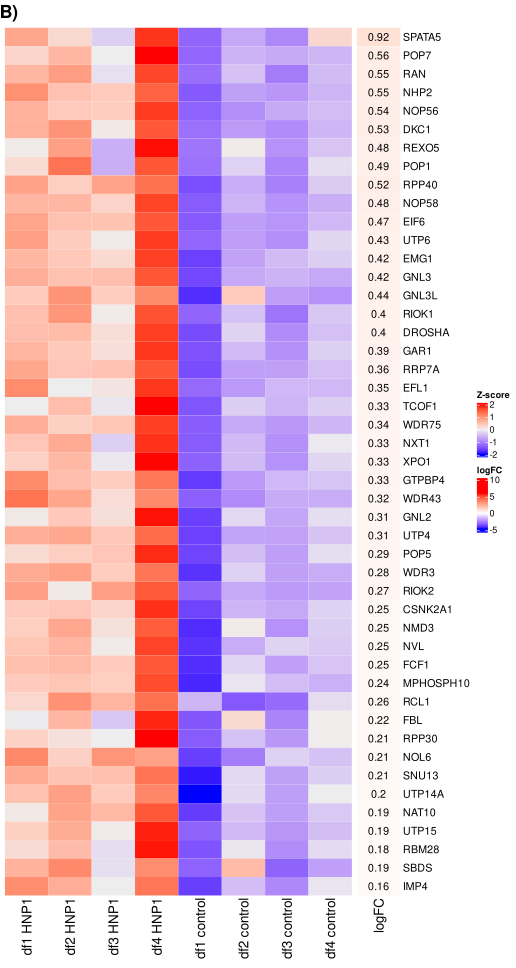

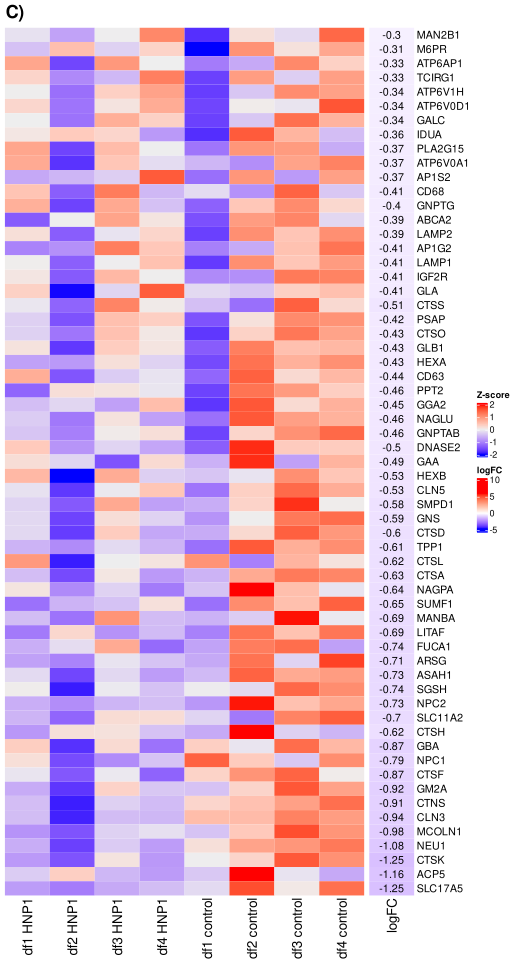


**Supplementary Figure 4.** Examples of expression heatmaps of upregulated pathways; **(a)** cell cycle and **(b)** ribosome biogenesis in eukaryotes, or a downregulated pathway **(c)** lysosome plotted to further visualize gene expression level in each sample. (Note: genes of df3 in cell cycle had relatively low expression level when compared to others but still showed upregulation when treated with HNP1. Heat map images were generated using ComplexHeatmap package version 2.16.0 (<https://bioconductor.org/packages/release/bioc/html/ComplexHeatmap.html>) in R version 4.3.1

**
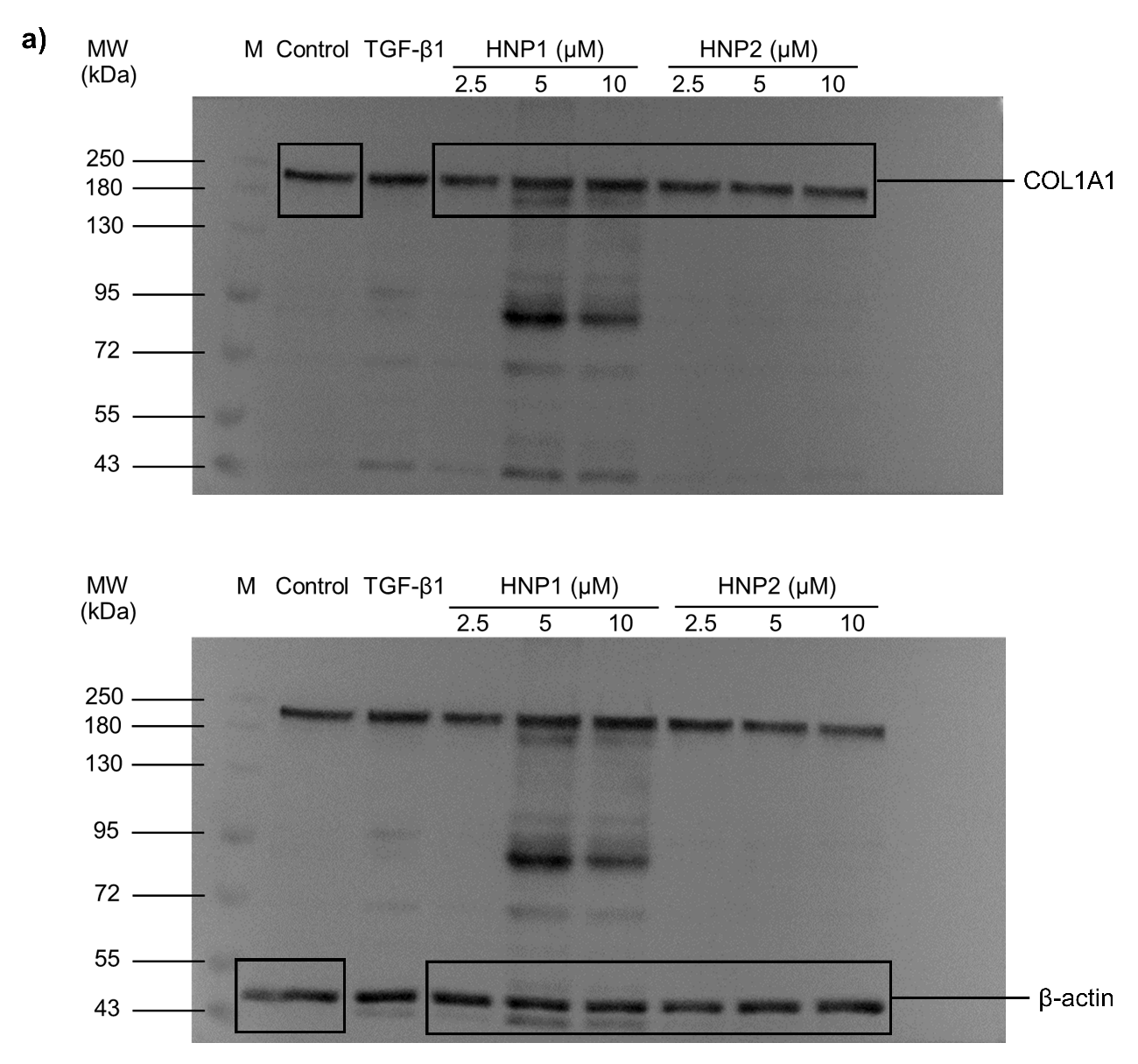
**

**
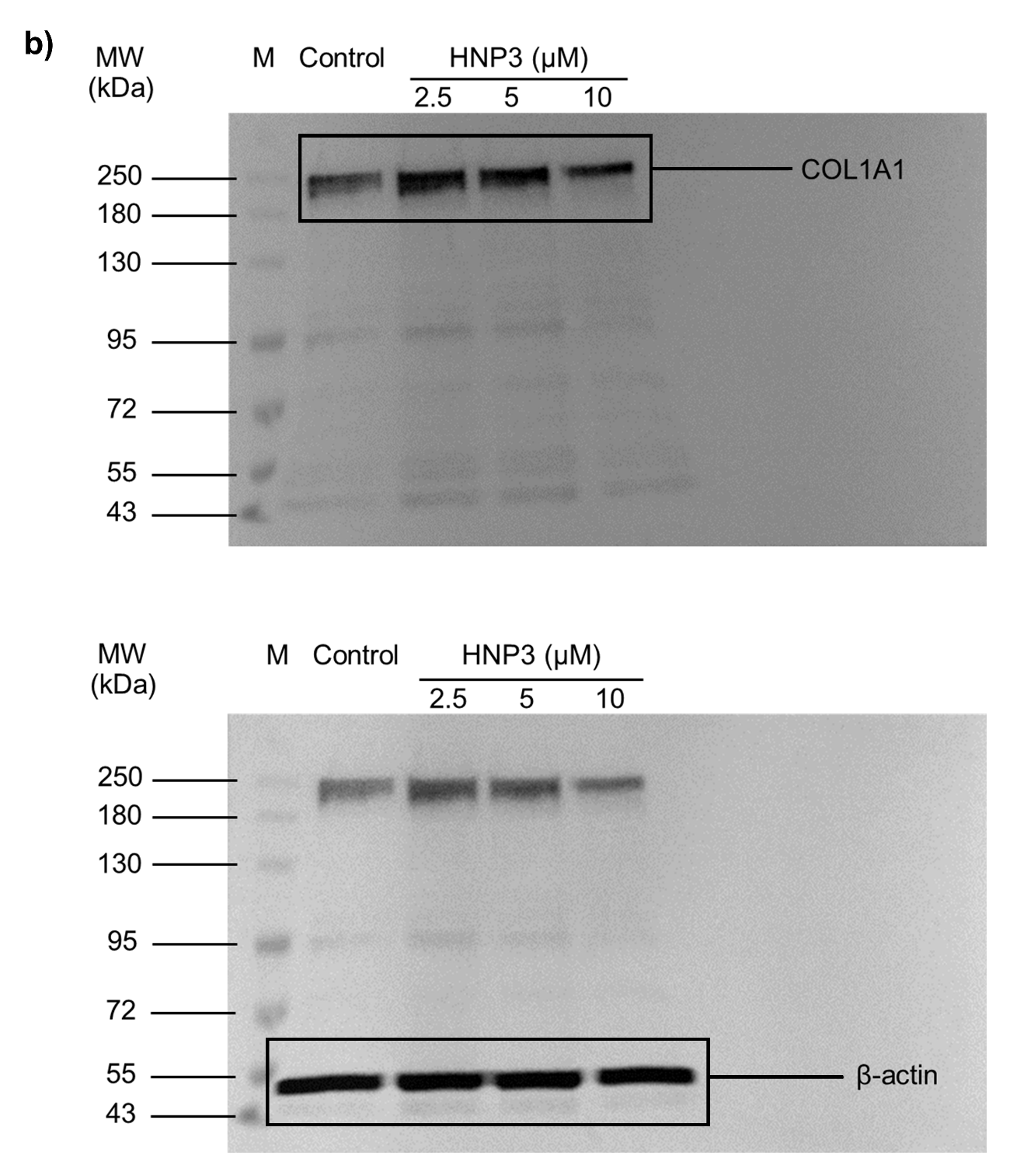
**

**Supplementary Figure 5.** Original images of Western blot analysis represent pictures for COL1A1 (molecular weight: 220 kDa) and β-actin (molecular weight: 45 kDa) protein expression by western blotting. β-actin is selected as an endogenous control. The black rectangle indicates the areas were cropped and shown in Figure 2. Bands in (a) were placed in Figure 2a and 2b, and bands in (b) were placed in Figure 2c. (Control as untreated, TGF- β1 as positive control and HNP1-3 at concentrations of 2.5, 5 and 10 μM).

**Supplementary Table 1.** The sequences of primers and probes for real-time PCR

| Genes | Forward primer (5’-3’) | Reverse primer (5’-3’) | Probes (5’-3’) |
| --- | --- | --- | --- |
| *COL1A1* | ACTGGCCCCCCTGGTCC | GGGCTCTCCAGCAGCACCTT | FAM-CCGGACCCCCAGGCCCACCT-TAMRA |
| *Ki-67* | ATTGAACCTGCGGAAGAGCTGA | GGAGCGCAGGGATATTCCCTTA | FAM-ACGACATGAAAACCAACAAAGA-TAMRA |
| *ABL* | TGGAGATAACATCTAAGCATAACTAAAGGT | GATGTAGTTGCTTGGGACCA | FAM-CCATTTTTGGTTTGGGCTTCACACCATT-TAMRA |

**Supplementary Table 2.** Number of reads and results of mapping to reference transcriptome with Salmon (n=4)

| Sample | Total reads (million) | Mapped reads (million) | Mapped rates (%) |
| --- | --- | --- | --- |
| df1 control | 16.46 | 15.38 | 93.45 |
| df2 control | 23.64 | 21.92 | 92.72 |
| df3 control | 21.76 | 20.10 | 92.36 |
| df4 control | 24.92 | 23.10 | 92.68 |
| df1 HNP1 | 26.98 | 25.31 | 93.81 |
| df2 HNP1 | 25.39 | 23.62 | 93.04 |
| df3 HNP1 | 25.59 | 23.78 | 92.93 |
| df4 HNP1 | 31.97 | 29.71 | 92.93 |

**Supplementary Table 3.** KEGG pathways with top-50 regulated DEGs (computed with pair fold-change value) are in bold character.

| A. Up-regulated |  | | |
| --- | --- | --- | --- |
|  |  |  |  |
| Pathway ID | Name | Count | Genes |
| hsa04115 | p53 signaling pathway | 19 | APAF1,CASP3,CCNB1,CCNB2,CCND1,CCND3,CCNE2,CDK1,CDK2,CDK6,CHEK1,CYCS,GADD45A,GADD45B,GTSE1,PMAIP1,**RRM2**,**SERPINE1**,SESN2 |
| hsa01230 | Biosynthesis of amino acids | 20 | ASL,**ASNS**,BCAT1,CBS,CPS1,CTH,ENO1,GOT1,GPT2,PFKP,PGAM1,PGK1,PHGDH,PRPS1,**PSAT1**,PSPH,PYCR1,PYCR2,SHMT2,TPI1 |
| hsa03460 | Fanconi anemia pathway | 24 | BLM,BRCA1,BRCA2,BRIP1,CENPX,EME1,FAAP24,FANCA,**FANCB**,FANCC,FANCD2,FANCG,FANCI,FANCL,FANCM,HES1,MUS81,RAD51,RMI1,RMI2,RPA3,SLX4,UBE2T,USP1 |
| hsa04110 | Cell Cycle | 44 | BUB1,BUB1B,BUB3,CCNA2,CCNB1,CCNB2,CCND1,CCND2,CCNE2,CDC20,CDC25A,CDC25C,**CDC45**,CDC6,CDC7,CDK1,CDK2,CDKN2D,DBF4,E2F1,E2F2,ESPL1,GADD45A,GADD45B,MAD2L1,MCM2,MCM3,MCM4,MCM5,MCM6,MCM7,MYC,ORC1,ORC3,ORC6,PCNA,PKMYT1,PLK1,PTTG1,RBL1,SKP2,SMC1A,TTK,YWHAH |
| B. Down-regulated |  | | |
|  |  |  |  |
| Pathway ID | Name | Count | Genes |
| hsa04913 | Ovarian steroidogenesis | 9 | ADCY4,ADCY9,**AKR1C3**,**CYP1B1**,IGF1R,PLA2G4A,**PLA2G4C**, **PTGS2**,STAR |
| hsa00982 | Drug metabolism | 14 | **ADH1B**,ADH5,ALDH3B1,CYP3A5,FMO4,GSTA4,GSTM2,GSTM3,GSTM4,GSTM5,MAOA,MGST1,MGST2,MGST3 |
| hsa04060 | Cytokine-cytokine receptor interaction | 57 | ACKR3,ACKR4,ACVR2B,BMP2,BMP4,BMP6,CCL2,CCL28,CCL7,CCL8,CCR10,CSF3,CTF1,CXCL1,CXCL10,CXCL12,CXCL14,CXCL2,CXCL3,CXCL5,CXCL6,CXCL8,EDA2R,GDF11,GDF15,GDF7,IFNAR1,IFNAR2,IFNGR1,IFNGR2,IL10RB,IL13RA1,IL13RA2,IL16, IL17D,IL1A,IL1B,IL1R1,IL1RL2,**IL1RN**,IL27RA,IL33,IL34,IL6,IL6R,LEPR,LIF,LTBR,TGFB3,TNFRSF10C,TNFRSF10D,TNFRSF11B,TNFRSF14,TNFRSF1B,TNFRSF21,TNFSF12,TNFSF13 |
| hsa04668 | TNF signaling pathway | 29 | CCL2,CEBPB,CXCL1,CXCL10,CXCL2,CXCL3,CXCL5,EDN1,ICAM1,IL1B,IL6,JUNB,LIF,MAP2K3,MAP2K6,MAP3K5,MAP3K8,MAPK3,MMP14,MMP3,NFKB1,NFKBIA,PIK3R2,PIK3R3,**PTGS2**,RPS6KA5,SOCS3,TNFAIP3,TNFRSF1B |
| hsa04920 | Adipocyte signaling pathway | 15 | ACACB,ACSL4,CD36,**CPT1B**,CPT1C,IRS2,LEPR,NFKB1,NFKBIA,NFKBIE,PPARA,PPARGC1A,RXRA,STAT3,TNFRSF1B |
